# Detection of Telomerase Reverse Transcriptase mRNA in Peripheral Blood Mononuclear Cells of Patients With Liver Failure

**DOI:** 10.5812/hepatmon.17976

**Published:** 2014-04-14

**Authors:** Zhu Chuanwu, Qian Feng, Li Ming, Wang Haiyan, Fang Huan, Luo Xiangrong, Zhang Xuehua, Zhu Xiang, Shen Xiujuan, Xu Ping

**Affiliations:** 1 Department of Hepatology, The Affiliated Infectious Disease Hospital of Soochow University, Suzhou, China; 2 Department of Infectious Diseases, The Second Affiliated Hospital of Bengbu Medical College, Bengbu, China; 3 Key Laboratory of Infection and Immunity, The Affiliated Infectious Disease Hospital of Soochow University, Suzhou, China

**Keywords:** Telomerase, Leukocytes, Mononuclear, Humans, Liver Failure, Prognosis

## Abstract

**Background:**

Telomerase activity is closely associated with the expression of human telomerase reverse transcriptase (*hTERT*) mRNA; although it can be induced in hepatocytes during liver regeneration, its dynamic change in patients with liver failure has remained unclear.

**Objectives:**

We investigated the variation and significance of *hTERT* mRNA expression in peripheral blood mononuclear cells (PBMCs) of the patients with liver failure.

**Patients and Methods:**

In this clinical experimental study, 76 Chinese patients were enrolled in the study between 2010 and 2012. The level of PBMCs *hTERT* mRNA was detected by relative quantitative real-time polymerase chain reaction (RT-PCR) in the samples taken before treatment and at seven-day intervals during a 28-day treatment period. The patients were divided into survivor and non-survivor groups according to the 3-months mortality after treatment. The dynamic variation of PBMCs *hTERT* mRNA was analyzed and its association with prognosis was assessed by the area under the receiver-operating characteristic curve.

**Results:**

The median level of PBMCs *hTERT* mRNA in survivors increased with treatment time and was significantly higher than the corresponding level in non-survivors after 14 days of treatment (P < 0.001). Subgroup analyses showed that the levels of PBMCs *hTERT* mRNA were remarkably higher in patients with acute-on-chronic liver failure than in those with chronic liver failure (P < 0.05). In patients with the same clinical type of liver failure, the level was markedly higher in survivors than in non-survivors after 14 days of treatment (P < 0.05); however, the levels were not significantly different between subgroups with different clinical type but the same prognosis. The sensitivity and specificity of PBMCs *hTERT* mRNA was high in evaluating the prognosis at day 14 and became much higher at days 21 and 28 post treatment. The expression of PBMCs *hTERT* mRNA had high sensitivity and specificity in evaluating the prognosis as early as day 14 post treatment and was significantly superior to the prognostic value of serum alpha-fetoprotein.

**Conclusions:**

The expression of PBMCs *hTERT* mRNA is closely associated with patient outcome, which indicates that *hTERT* mRNA in PBMCs might be useful as a prognostic biomarker of liver failure.

## 1. Background

Telomerase, an RNA-dependent DNA polymerase, can maintain telomeric length and therefore, make the cell achieve unlimited proliferation capacity (1). Telomerase consists of two subunits, namely the functional RNA component and the human telomerase reverse transcriptase (*hTERT*) catalytic subunit (2, 3). The expression of the mRNA encoding *hTERT* closely reflects the telomerase activity (4, 5). In contrary to normal somatic cells, germ cells, stem cells, and tumor cells often express higher level of telomerase activity (6, 7). It indicates that telomerase expression is closely associated with cell differentiation and development. A significantly increased telomerase activity in peripheral blood or peripheral blood mononuclear cells (PBMCs) has been observed in patients with hepatocellular carcinoma (HCC) (8, 9), which suggests a close association between the activation of telomerase and the tumorigenesis of the liver.

The proliferation of hepatocytes is usually a common physiological process in regeneration of both hepatic precursor cells and quiescent hepatocytes in response to liver injury; however, little is known about the telomerase activity in normal hepatocyte growth. The decline in regenerative capacity of the liver in patients with cirrhosis was associated with telomere shortening and hepatocyte senescence (10). A low expression of *hTERT* mRNA was detected in PBMCs of patients with chronic hepatitis B and C (11, 12). Moreover, a significantly lower serum *hTERT* mRNA was found in fulminant hepatitis in comparison to acute hepatitis and severe acute hepatitis (13). In animal experimental research, the telomere dysfunction in telomerase-deficient mice was associated with defects in liver regeneration and acceleration of developing liver cirrhosis in response to chronic liver injury (14). A recent report has revealed that *hTERT* is induced in hepatocytes during liver regeneration (15). All these studies indicated that telomerase might be involved in the pathogenesis of liver diseases.

The prognosis of patients with severe liver injury, especially with liver failure, largely depends upon the regenerative capacity of hepatocytes during the comprehensive treatment. In clinical practice, serum alpha-fetoprotein (AFP) is usually used as a predictive biomarker for monitoring the prognosis of patients with liver failure because it reflects the regeneration of hepatocytes in response to liver injury (16-18). Although the most important function of telomerase is associated with cell proliferation and regeneration, yet, there is no report concerning the association of the liver failure prognosis and telomerase activation. 

## 2. Objectives

In the present study, we investigated the dynamic change of *hTERT* mRNA expression in PBMCs of patients with liver failure as well as the predictive value of PBMCs *hTERT* mRNA in the liver failure prognosis. Additionally, we compared its prognostic value with serum AFP.

## 3. Patients and Methods

### 3.1. Study Subjects

This was a clinical experimental study. The patients who were hospitalized in Department of Hepatology, the Affiliated Infectious Disease Hospital of Soochow University, China, between November 2010 and December 2012 and met the diagnostic criteria for liver failure issued by Chinese liver failure and artificial liver group et al. (19) were included. The patients diagnosed with sub-acute liver failure (SALF), acute-on-chronic liver failure (ACLF), and chronic liver failure (CLF) were enrolled in the study. Those diagnosed with acute liver failure (ALF) or with multiple system organ failure (MSOF) were excluded. In addition, the patients with simultaneous HCC or non-hepatic tumor, HIV infection, or malignant hematological disease were excluded. During this study period, 45 patients with liver failure were excluded. Healthy control group consisted of our 10 volunteer colleagues, 6 males and 4 females with the average age of 33.9 years (ranging from 25 to 45 years of age). Our hospital ethics committee approved the study (Code: szst-p-2010-2, September 2010) and written informed consent was obtained from each patient.

### 3.2. Therapeutic Regime

All patients received comprehensive treatment after admission to the hospital. The basic treatment measures were taken for each patient including resting in bed, complement of energetic contents and vitamins, maintaining fluid, electrolyte and acid-base homeostasis, and treatment and/or prevention of complications. The medicines such as glycyrrhizic acid injection, glutathione, polyene phosphatidyl choline, prostaglandin E1, and S-adenosylmethionine were prescribed to alleviate hepatocyte inflammation and necrosis. Albumin (ALB) and fresh plasma were intermittently transfused. On the basis of above medical treatment, the vast majority of patients were given artificial liver support system (ALSS) treatment; most of the patients were treated with plasma exchange and a small number of cases with plasma bilirubin adsorption or albumin dialysis (MARS) due to the shortage of fresh plasma. In addition, oral antiviral therapy was administered to those with vigorous hepatitis B virus (HBV) infections (serum HBV DNA level > 4 log10 IU/mL). 

### 3.3. Routine Laboratory Tests

The routine serum biochemical tests were ordered for all the patients after admission and during treatment. These tests included serum alanine aminotransferase (ALT), total bilirubin (TBIL), ALB, international normalized ratio (INR) of blood coagulation, prothrombin time (PT), prothrombin activity (PTA), creatinine (Cr), and electrolytes (Hitachi Model 7600 Series Automatic Analyzer, Japan). Viral hepatitis markers for hepatitis A, B, C, Delta, and E viruses (ARCHITECT i2000SR, Abbott Laboratories Ltd, USA) as well as the antibodies for autoimmune liver diseases were examined. At the same time, HBV DNA levels were quantitatively tested for patients with positive serum hepatitis B surface antigen (HBsAg) (LightCycler 480 System, Roche, Penzberg, Germany). The serum AFP was respectively examined in all patients during the first seven days and after 14-28 days of treatment by electrochemiluminescence assay (Roche Cobas E 601).

### 3.4. Specimen Collection and Separation of Peripheral Blood Mononuclear Cells 

Five milliliters of EDTA-anticoagulated peripheral blood was drawn from each patient on admission (before treatment), at days 7, 14, 21, and 28 during treatment. PBMCs were isolated by standard Ficoll density-gradient centrifugation and then were washed with 0.01 M phosphate buffer solution (PBS). After centrifugation, the cells were dissolved in 20 µL of PBS and kept frozen at -80 until the samples were used.

### 3.5. RNA Extraction and Relative Quantitative Real-time Polymerase Chain Reaction

Total cellular RNA was extracted using BIOZOL (Hangzhou Bioer Technology Co. Ltd, Hangzhou, China) according to the manufacturer’s instruction. Briefly, the samples of PBMCs were respectively precipitated and lysed; the cellular RNA from each sample was isolated, precipitated, washed, and dissolved in 50 µL of RNase-free water. The integrity of RNA was determined for each sample. Relative quantitative real-time polymerase chain reaction (RT-PCR) was employed with an AB7300 cycler (Applied Biosystems, Carlsbad, CA, USA). For the reverse transcription (RT) step, cDNA was synthesized in a total volume of 10 µL containing 5 µL of RNA using the PrimeScript RT regent kit (TaKaRa Biotecnology Co. Ltd, Dalian, China). The reactions were incubated in the thermocycler in thin-walled 0.2 ml PCR tubes for 15 min at 37 °C, 5 s at 85 °C, and then held at 4 °C. After RT finished, SYBR Premix DimerEraser™ with Rox PCR Master Mix (TaKaRa Biotecnology Co. Ltd, Dalian, China) was employed to amplify the corresponding genes with primers specific for *hTERT* (forward: 5ʹ-TGACACCTCACCTCACCCAC-3ʹ; reverse:5ʹ-CACTGTCTTCCGCAAGTTCAC-3ʹ) and for human β-actin (internal control gene, forward: 5ʹ-TGAGCGCGGCTACAGCTT-3ʹ; reverse: 5ʹ-TCCTTAATGTCACGCACGATTT-3ʹ), which was used as a housekeeping gene. RT-PCR settings were as follows: at 95 °C for 30 s, followed by 40 cycles at 95 °C for 5 s, at 60 °C for 34 s, 95 °C for 15 s, at 60 °C for 1 min, and at 95 °C for 15 s, consecutively, according to the manufacturer’s protocol. Relative quantification was performed using the comparative threshold (CT) method (ΔΔCT, delta-delta CT) after determining the CT values for target gene (*hTERT*) and internal control gene (β-actin) in each sample; the data were analyzed using the 2^-ΔΔCT^ method (20). Fold changes in target mRNA expression level were calculated after normalization to β-actin. A calibrator sample was the one from arbitrarily selected healthy control sample. The ΔΔCT method provides a relative quantification ratio according to calibrator that allows statistical comparisons of gene expression among samples. Changes in gene expression were reported as fold changes relative to control.

### 3.6. Statistical Analysis

All results were reported as mean ± SD or median (range). The normality of data distribution was tested by the Kolmogorov-Smirnov test. Pearson's chi-square test, independent-samples t test, nonparametric Mann-Whitney U or Wilcoxon signed ranks test, logistic regression, and nonparametric Spearman’s rank correlation were used when appropriate. Receiver operating characteristic (ROC) curves were generated to determine the areas under the ROC curves (AUC), their 95% confidence intervals (CI), and optimal cut-off values as well as its sensitivity and specificity for PBMCs *hTERT* mRNA or serum AFP. Data analysis was performed using SPSS version 16.0 (SPSS, Inc., Chicago, IL, USA). P value < 0.05 was considered statistically significant.

## 4. Results

### 4.1. Baseline Clinical Characteristics and Prognosis

During the 28-day study period, 76 patients with complete data were enrolled in the study. Among those three patients were diagnosed with SALF, 45 and 28 patients were diagnosed as ACLF and CLF, respectively, according to the classification of Chinese guidelines for liver failure (19). With regard to the etiology of the disease, 68 cases were caused by HBV, four by HBV and hepatitis delta virus co-infection, three by hepatitis E virus, and one by Chinese herbal medicine. During a three-month follow-up period after the start of treatment, 52 patients survived including one case who underwent liver transplantation after two months of treatment, and 24 patients died. Therefore, based on the three-month mortality, the patients were allocated into two groups: survivors and non-survivors. The two groups did not differ with regard to the baseline clinical characteristics (Table 1). The similar management protocol was administered to both groups. There were 43 patients in survivor group and 22 in non-survivor group who received ALSS treatment; the average number of times was 2.1 and 2.7 in survivors and non-survivors, respectively, which was not significantly different between two groups (t = 1.879, P = 0.064).

**Table 1.** Comparison of Baseline Characteristics in Patients With Different Clinical Outcomes ^a^, ^b^

Characteristic	Survivors (n = 52)	Non-survivors (n = 24)	Test Value	P Value
**Male**	41 (78.9)	17 (70.8)	0.583	0.445 ^c^
**Age, y**	46.6 ± 12.8	49.1 ± 14.9	0.758	0.751 ^d^
**Course of liver failure ** ^**d**^	11.5 ± 6.5	14.2 ± 6.8	1.624	0.109 ^d^
**ALT, IU/L **	98.5 (19-2114)	167.5 (29-1000)	568.0	0.531 ^e^
**TBIL, μmol/L**	319.2 ± 130.7	314.8 ± 141.6	0.132	0.895 ^d^
**ALB, g/L**	29.8 (21.8-43.6)	30.4 (21.6-42.0)	0.135	0.893 ^e^
**INR**	2.3 (1.6-4.2)	2.1 (1.5-10.3)	1.290	0.200 ^e^

^a^ Abbreviations: ALB, serum albumin; ALT, alanine aminotransferase; INR, international normalized ratio; TBIL, total bilirubin.

^b^ Data are presented in Mean ± SD, No. (%) and Median (range).

^c^ P value calculated using chi square test.

^d^ P value calculated using t test.

^e^ P value calculated using Mann-Whitney U test.

### 4.2. Peripheral Blood Mononuclear Cells hTERT mRNA Expression in Survivors and Non-Survivors

**Figure 1.**
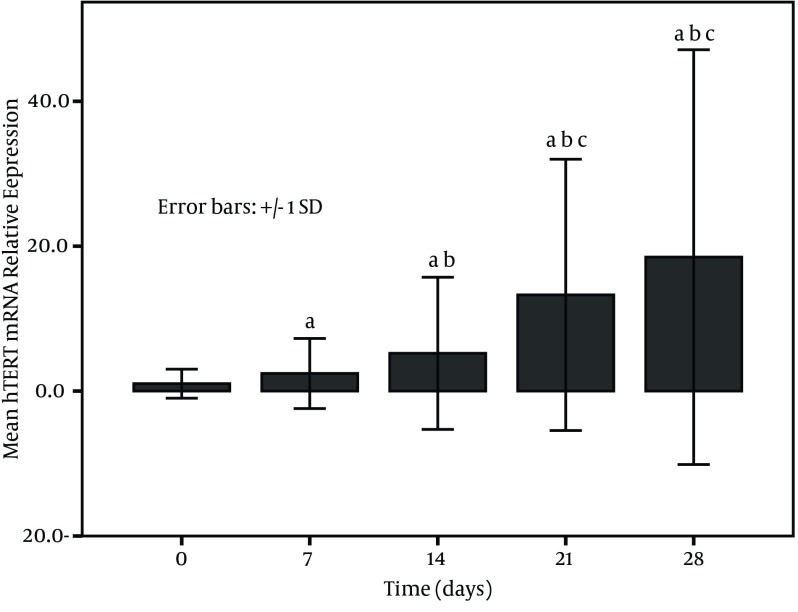
Mean Levels of Peripheral Blood Mononuclear Cells hTERT mRNA Relative Expression in 52 Survivors With Liver Failure a, versus day 0 (before treatment); b, versus day 7 (after 7 days of treatment); c, versus day 14 (after 14 days of treatment) (P < 0.001).

**Figure 2.**
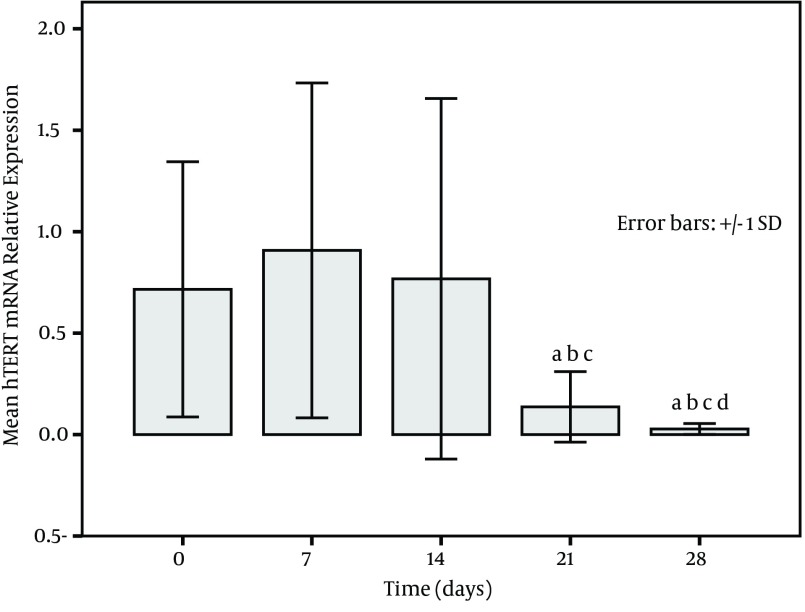
Mean Levels of Peripheral Blood Mononuclear Cells *hTERT* mRNA Relative Expression in 24 Non-Survivors With Liver Failure a, versus day 0 (before treatment); b, versus day 7 (after 7 days of treatment); c, versus day 14 (after 14 days of treatment); d, versus day 21 (after 21 days of treatment) (P < 0.001).

The expression of PBMCs *hTERT* mRNA increased with treatment time in survivors and became higher at post treatment days 21 and 28 (Figure 1). on the other hand, a low expression in non-survivors was seen at each time point (at baseline, at post treatment days 7, 14, 21, and 28) and especially the levels at post treatment days 21 and 28 were significantly decreased in comparison to the baseline (P < 0.001) (Figure 2). The relative levels of PBMCs *hTERT* mRNA expressed at post treatment days 14, 21, and 28 were significantly higher in survivors than in non-survivors at the corresponding time point (U-value was 299.0, 16.0, and 17.5, respectively; P < 0.001 in all). In addition, the median level in survivors was significantly lower than 2.959 (0.161-11.863) expressed in healthy individuals only at baseline and at post treatment day 7 (all P < 0.05); however, the level was dramatically lower in non-survivors than in healthy group at each study time point (all P < 0.01).

### 4.3. Comparison of Peripheral Blood Mononuclear Cells hTERT mRNA Expression between Patients with Different Clinical Types of Liver Failure

Logistic regression was used to analyze the association between patients' outcome and baseline clinical characteristics including age, gender, course of disease (days diagnosed with liver failure), clinical types of liver failure, ALT, TBIL, ALB, INR, Cr, and the number of times receiving ALSS treatment. We found that the clinical type of liver failure was an independent factor influencing the prognostic outcome (OR = 5.073, 95% CI = 1.663-15.480, P = 0.004). Further analysis showed that the prognosis was significantly different between patients with ACLF and CLF (OR = 0.134, 95% CI = 0.038-0.478, P = 0.002) and the expression of PBMCs *hTERT* mRNA was found to be remarkably different between ACLF and CLF subgroups at each study time point during a 28-day treatment period (Table 2).

**Table 2.** Median Levels of Peripheral Blood Mononuclear Cells *hTERT* mRNA in Patients With Different Clinical Type of Liver Failure ^a^, ^b^

	ACLF (n = 45)	CLF (n = 28)	U Value	P Value
**Before treatment**	0.414 (0.098-5.161)	0.442 (0.090-13.332)	579.0	0.563
**Post** **treatment**				
7 days	1.144 (0.068-33.489)	0.489 (0.071-6.048)	414.0	0.014
14 days	1.669 (0.023-63.149)	0.769 (0.025-7.554)	436.5	0.028
21 days	5.729 (0.020-78.472)	0.188 (0.012-77.450)	377.0	0.004
28 days	4.909 (0.005-138.180)	0.041 (0.004-116.739)	385.0	0.005

^a^ Abbreviations: ACLF, acute-on-chronic liver failure; CLF, chronic liver failure.

^b^ All values are Median (range).

### 4.4. Comparison of Peripheral Blood Mononuclear Cells hTERT mRNA Expression between Different Subgroups

Since there were only three patients in SALF subgroup, we did not analyze their data. Of 45 patients with ACLF, 38 survived and seven died and of 28 patients with CLF, 12 survived and 16 died. For patients with same clinical type of liver failure, the analysis showed that the levels of PBMCs *hTERT* mRNA relative expression in survivors at post treatment days 14, 21, and 28 were significantly higher than in non-survivors (all P < 0.05). However, for those with different clinical type but the same prognosis, the result showed that the levels of PBMCs *hTERT* mRNA expression had no significant difference at each study time point (P > 0.05).

### 4.5. Evaluation of Peripheral Blood Mononuclear Cells hTERT mRNA Expression in Determining Prognosis

The ROC curves for PBMCs *hTERT* mRNA relative expression at different time points and survival prognosis showed that the AUC was 0.615 (95% CI: 0.489-0.741, P > 0.05) at post treatment day seven, 0.760 (95% CI: 0.652-0.869, P < 0.001) at post treatment day 14, 0.987 (95% CI: 0.965-1.009, P < 0.001) at post treatment day 21, and 0.986 (95% CI: 0.958-1.014, P < 0.001) at post treatment day 28. For the optimal cut-off value of 0.408 at post treatment day 14, the predictive sensitivity was 76.9% and the specificity was 54.2%. For the cut-off value of 0.621 at post treatment day 21 and 0.209 at post treatment day 28, the predictive sensitivity rates were 96.2% and 98.1%, respectively, and the specificity of either of them was nearly 100%.

### 4.6. Comparison of Peripheral Blood Mononuclear Cells hTERT mRNA and Serum AFP in Evaluating Prognosis

The median levels of serum AFP within seven days post treatment in survivors and non-survivors was not significantly different (51.9 and 44.4 ng/mL, respectively; U = 535.5, P > 0.05), but the median level in survivors remarkably increased after 14-28 days of treatment in comparison to non-survivors (87.3 and 45.0 ng/mL, respectively; U = 403.5, P < 0.05). ROC curves for serum AFP and good prognosis revealed that the AUC was 0.571 (95% CI: 0.425-0.717, P > 0.05) within seven days of treatment and 0.677 (95% CI: 0.549-0.805, P = 0.014) after 14-28 days of treatment. The results indicated that the clinical outcome was significantly associated with elevated level of serum AFP after at least 14 days of treatment. At the optimal cut-off value of 49.5 ng/mL of serum AFP after 14-28 days of treatment, the predictive sensitivity was 67.3% and the specificity was 70.8%.

**Figure 3.**
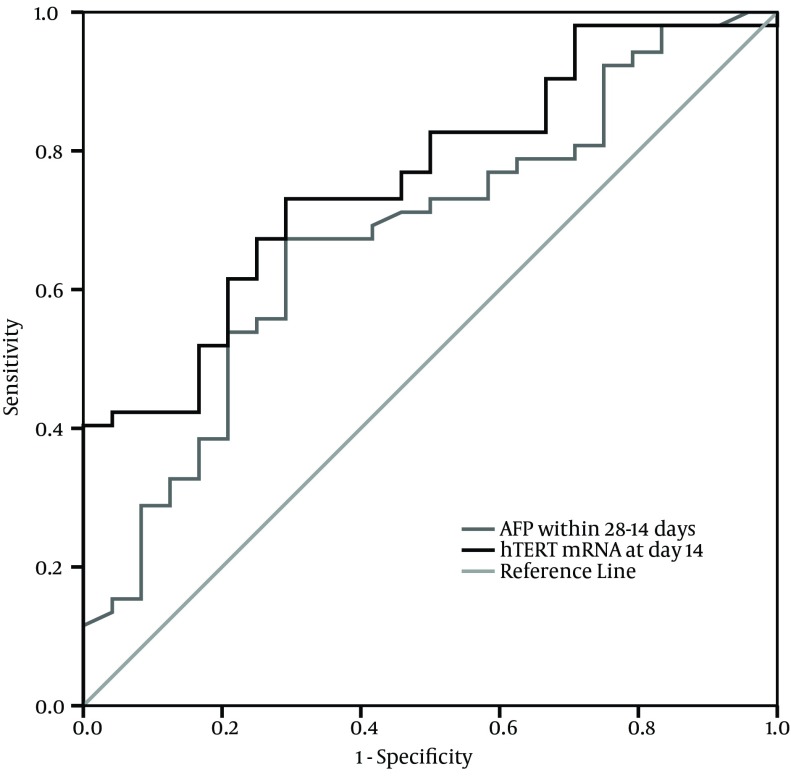
Receiver Operating Characteristic Curves for Good Prognosis by Peripheral Blood Mononuclear Cells *hTERT* mRNA Expression and Serum AFP

We analyzed the association between serum AFP level within 14-28 days after treatment and PBMCs *hTERT* mRNA expression at post treatment day 14 and we found a significantly positive correlation between the two indicators (r = 0.844, P < 0.001). The comparison of ROC curves showed that PBMCs *hTERT* mRNA at post treatment day 14 had better prognostic value than serum AFP within 14-28 days after treatment (Figure 3).

## 5. Discussion

Liver failure is a complex medical emergency that evolves after a catastrophic insult to the liver and its outcome is still the most ominous of all gastroenterologic diseases. Serious complications tend to occur in the course of the disease and further exacerbate the problems. The prognosis mainly depends on the control of inflammation and necrosis of the liver, prevention or treatment of the complications that may arise, and regeneration of hepatocytes during treatment. Based on medical management, the use of ALSS will benefit the survival rate; however, when spontaneous survival is considered possible, liver transplantation is unlikely considered as the last therapeutic option.

It is a routine practice to assess the prognosis of the disease during treatment. The prognostic indices include age, INR, PTA, TBIL, hepatic encephalopathy, AFP, MELD score, and amino acid metabolism and so on (17, 21-24); however, there is not a specific indicator to reflect the real situation of liver regeneration yet. The recovery of necrotic liver is closely related to the proliferation and regeneration of hepatocytes. It is possibly associated with telomerase activity because telomerase is a kind of enzyme involving in cell differentiation and proliferation. Sirma et al. (15) found that *hTERT* in hepatocytes was induced during liver regeneration in vivo and hepatocyte proliferation in vitro. Telomerase activity in PBMCs is closely linked to its expression in the liver in patients with HCC (8, 9). In this study, we found that the levels of PBMCs *hTERT* mRNA expression were very low in patients with liver failure before treatment, and the levels were not significantly different between survivors and non-survivors during the early days of treatment. The result is partly similar to the findings of Miura et al. (13), which showed that the level of serum *hTERT* mRNA was lowest in fulminant hepatitis, and was also significantly lower than that in acute hepatitis. However, we found that the median levels in survivors increased with treatment time while those were all lowly expressed in non-survivors during the study period. The results strongly indicated that a patient with an increasing expression of PBMCs *hTERT* mRNA during treatment might have a good prognosis.

Among the clinical indices that might influence the outcome, logistic regression analyses showed that the clinical type of liver failure was an independent factor; the survival rate of patients with ACLF was significantly higher than that of patients with CLF (84.4% [38/45] vs. 42.9% [12/28], P < 0.001). Further analysis demonstrated that the median level of PBMCs *hTERT* mRNA in patients with ACLF was markedly higher than in patients with CLF. To some extent, this was in accordance with the fact that the hepatocyte proliferation is likely more vigorous in patients with ACLF than with CLF. The reason may partly be due to the difference of hepatic fibrosis or cirrhosis between these two clinical types of liver failure. According to the criteria of liver failure classification in Chinese guideline (19), ACLF occurs in patients with the background of chronic liver diseases while CLF evolves in patients with liver cirrhosis.

In patients with the same clinical type of liver failure, a similar result was found; the median levels of PBMCs *hTERT* mRNA expression after 14 days of treatment were significantly higher in survivors than in non-survivors subgroup with ACLF and CLF. In those with different clinical type but the same prognosis, the median level at each study time point was not significantly different between the two subgroups. These results further elucidated the close association between the expression of PBMCs *hTERT* mRNA and the prognosis of liver failure. Therefore, the monitoring of *hTERT* mRNA expression during treatment of liver failure may help to evaluate the status of hepatocyte regeneration. If the detected sample is liver tissue instead of PBMCs, the result may directly reflect the real situation of liver regeneration; however, to perform liver biopsy for these patients is not feasible. Serum *hTERT* mRNA can be detected but the concentration may be disturbed by plasma exchange treatment; on the other hand, the sample from PBMCs is considerably stable and is not easily influenced by endogenous and exogenous factors. Previous studies have shown the involvement of Kupffer cells, resident NK cells, T lymphocytes, interferon-α/-γ, and colony stimulating factor during liver regeneration (25-29); therefore, our findings indicated that PBMCs were associated with liver regeneration.

Considering the value of PBMCs *hTERT* mRNA in evaluating the prognosis of liver failure, our results showed that the AUC of PBMCs *hTERT* mRNA at day 14 had a high predictive value to identify good prognosis and had a much higher sensitivity as well as specificity to predict the outcome with treatment time. It suggested that the monitoring of PBMCs *hTERT* mRNA expression could effectively evaluate the prognosis. In addition, our data showed a positive correlation between the levels of serum AFP within 14-28 days after treatment and PBMCs *hTERT* mRNA expression at post treatment day 14; this consistent relation further revealed the intrinsic association between liver regeneration and serum AFP as well as PBMCs *hTERT* mRNA. However, the AUC of PBMCs *hTERT* mRNA at post treatment day 14 had higher prognostic value than serum AFP within 14-28 days after treatment. This is somewhat similar to a previous study that showed serum *hTERT* mRNA was superior to serum AFP in diagnosis of HCC (30).

In conclusion, in this preliminary study, we report for the first time that the expression of PBMCs *hTERT* mRNA was associated with the prognosis of liver failure; therefore, *hTERT* mRNA in PBMCs might become a promising candidate as a biomarker for evaluating the outcome of liver failure. Low expression of PBMCs *hTERT* mRNA through the course of the disease may indicate a bad liver regeneration and a poor prognosis; thus early liver transplantation should be considered in order to improve survival of patients who are incapable of responding to medical treatment. Obviously, this study was just preliminary, the sample size was not large, and the expression of PBMCs *hTERT* mRNA was detected by relatively quantitative RT-PCR. Hence, further studies with larger sample size and quantitative RT-PCR assay are needed to confirm our findings. At the same time, the intrinsic mechanism between PBMCs *hTERT* mRNA and liver regeneration remains to be elucidated.
